# Host Serum Proteins as Potential Biomarkers of Bovine Tuberculosis Resistance Phenotype

**DOI:** 10.3389/fvets.2021.734087

**Published:** 2021-11-18

**Authors:** Jorge Luis Mazorra-Carrillo, Omar Antonio Alcaraz-López, Gonzalo López-Rincón, Bernardo Villarreal-Ramos, José A. Gutiérrez-Pabello, Hugo Esquivel-Solís

**Affiliations:** ^1^Biotecnología Médica y Farmacéutica, Centro de Investigación y Asistencia en Tecnología y Diseño del Estado de Jalisco, Guadalajara, Mexico; ^2^Laboratorio de Investigación en Tuberculosis Bovina, Facultad de Medicina Veterinaria y Zootecnia, Universidad Nacional Autónoma de México, Mexico City, Mexico; ^3^Department of Bacteriology, Animal and Plant Health Agency, Addlestone, United Kingdom; ^4^Centre of Excellence for Bovine Tuberculosis, Institute for Biological, Environmental and Rural Sciences, Aberystwyth University, Aberystwyth, United Kingdom

**Keywords:** host resistance to infection, *Mycobacterium bovis*, bovine tuberculosis, biomarkers, proteome, ELISA, cattle

## Abstract

Eradication of bovine tuberculosis (bTB) continues to be a worldwide challenge. The lack of reliable vaccines dampens the control and eradication programs of *Mycobacterium bovis* infection and spread. Selection and breeding of cattle resistant to *M. bovis* infection would greatly enhance the effectiveness of bTB eradication programs. Here, we have evaluated the potential of serum proteins as biomarkers of cattle resistance to bTB in Holstein-Friesian cows, 6–8-year-old, born and raised in similar conditions in herds with bTB prevalence >30%. Serum proteins obtained from uninfected cows (bTB-resistant; R) were compared to those from infected cows (bTB-susceptible; S), defined by a negative or positive bTB diagnosis, respectively. bTB diagnosis included: (i) single intradermal (caudal fold) tuberculin test, (ii) whole blood IFN-gamma test, (iii) gross visible lesions in lymph nodes and lungs by inspection at the abattoir, and (iv) a bacteriological culture for *M. bovis*. Using 2D-GE and LC-ESI-MS/MS, we found higher expression levels of primary amine oxidase (AO), complement component 5 (C5), and serotransferrin (TF) in R cattle than S cattle. In-house developed and standardized ELISAs for these novel biomarkers showed the best sensitivities of 72, 77, 77%, and specificities of 94, 94, 83%, for AO, C5, and TF, respectively. AUC-ROC (95% CI) values of 0.8935 (0.7906–0.9964), 0.9290 (0.8484–1.010), and 0.8580 (0.7291–0.9869) were obtained at cut-off points of 192.0, 176.5 ng/ml, and 2.1 mg/ml for AO, C5, and TF, respectively. These proteins are involved in inflammatory/immunomodulatory responses to infections and may provide a novel avenue of research to determine the mechanisms of protection against bTB. Overall, our results indicate that these proteins could be novel biomarkers to help identify cattle resistant to bTB, which in turn could be used to strengthen the effectiveness of existing eradication programs against bTB.

## Introduction

Members of the *Mycobacterium tuberculosis* complex are a group of pathogenic mycobacteria responsible for tuberculosis (TB) in mammals. The principal causative agent of bovine TB (bTB) is *Mycobacterium bovis*, which affects the livestock industry worldwide (1). bTB is an ancient animal disease that represents a zoonotic public health concern (2). While test and slaughter programs using the tuberculin skin test have led to the eradication of bTB in several countries, bTB remains endemic in some geographic areas (3). In addition to the negative impact of bTB on livestock productivity, the surveillance program is costly and has placed an extra economic burden on farmers and governments (4). Moreover, the test-and-slaughter program is not compulsory in all countries, and in countries where this program is voluntary, the extra cost and the time it takes to eradicate the disease from the herd causes farmers to lose interest and withdraw from the program. Alongside is the pressure of other domesticated and wild animals infected with *M. bovis* that pose the ability to infect bTB-free cattle herds (5).

Currently, there is no licensed vaccine against bTB; the leading candidate is the live attenuated *M. bovis* bacilli Calmette-Guerin (BCG); however, BCG is not currently used as the protection it confers is variable and interferes with the current diagnostic tests (6). Therefore, new interventions are needed which may enhance the effectiveness of bTB eradication programs, which could eventually lead to the elimination of bTB. In this context, increasing the genetic resistance of cattle to bTB could help control bTB by reducing the susceptibility to infection of animals and by potentially allowing infected animals to contain the infection and therefore reducing environmental contamination (7). Genetic or natural disease resistance is the inherent capacity of an animal, involving both immune and non-immune mechanisms, to resist disease when exposed to pathogens (8). Natural resistance to bTB in cattle has been phenotypically observed (nonreaction to bTB tests, absence of lesions and negative *M. bovis* culture) and extensively evaluated in families and breeds exposed to *M. bovis* under field conditions (9–13). Based on bTB phenotypes, robust heritability estimates of genetic resistance to *M. bovis* in cattle has been statistically estimated over time and in different climatic zones, indicating that breeding for increased bTB resistance in cattle is a feasible strategy (9–15).

Implementing immunogenomics with genome editing for the generation of transgenic-resistant cattle to bTB is currently being tested as a promising strategy (16–19). Genetically modified cows with enhanced anti-mycobacterial capacity have been generated by insertion of the mouse intracellular pathogen resistance 1 gene (*Ipr1*), named as the Sp110 nuclear body protein (*SP110*), through transcription activator-like effector nuclease (TALEN) (16). In comparison, the insertion of the human defensin β-3 gene (*DEFB103A*) through plasmid transfection and somatic cell nuclear transfer has also achieved bTB-resistant cattle (17). The knock-in genome editing with the bovine gene natural resistance-associated macrophage protein-1 (NRAMP1), renamed as the functional solute carrier family 11A member 1 gene (*SLC11A1*), has produced cattle with increased resistance to bovine tuberculosis (18, 19). *SLC11A1* gene-edited bTB-resistant cattle have been successfully produced through the single clustered regulatory interspaced short palindromic repeats (CRISPR)-associated protein 9 (Cas9) nickase (Cas9n) and somatic cell nuclear transfer (18); system that has been improved implementing the homology-mediated end-joining (HMEJ)-based method (19). NRAMP1 is a divalent metal ion transporter whose expression occurring only in macrophages and other phagocytes is upregulated by cytokines and induces iron sequestration and the production of nitric oxide (NO), decreasing the survival of *M. bovis* and other intracellular pathogens (20, 21). The overexpression of bovine NRAMP1 provides cattle with improved resistance to bTB (18, 21). Polymorphisms of the *SLC11A1* gene influencing the NRAMP1 expression have been related to bTB-resistance in African Zebu cattle (22) and Chinese Holstein cattle (23).

*M. bovis* infects, resides, and replicates in monocytes-derived cells of infected cattle. Therefore, immunogenetic studies on those cells have been of particular interest for searching bTB-resistance candidate genes (24). Genomic microarray analysis in cDNA isolated from naïve bovine macrophages has identified the interleukin-1 receptor antagonist (IL1RN), a good candidate biomarker of bTB resistance of Mexican dairy Holstein-Friesian cattle (25). Genome-wide association studies (GWAS) in case-control matches have previously identified new SNPs and QTL regions associated with bTB resistance/susceptibility in dairy cattle from Mexico (10), Ireland (26, 27), UK (11, 14), and Cameroon (12). GWAS also revealed that bTB resistance is polygenic (12) and has no heterozygote advantage (28). Like genome analysis, serum proteomics is another reliable approach to predict biomarkers of bTB-resistance. Preliminary findings on the levels of serum proteins in cattle and their correlation with degrees of mycobacterial infection have led to the discrimination of clinical and subclinical bTB and different stages of bovine paratuberculosis (Johne's disease) from not infected cattle and exposed cattle (29–32). Furthermore, analyses of concentration in serum of proteins that impact the immune system and macrophage function, i.e., adiponectin, ceruloplasmin, and conglutinin (33–35), have revealed that those are under genetic control and heritability (36–38), and a negative association with the predisposition of respiratory infectious diseases in cattle (36, 37, 39) with implications in bTB (35). These findings suggest that identifying serum proteins for resistance against bTB is possible in cattle and since non-specific serum proteins have a role in protective immunity, measuring the level of serum proteins may be a helpful trait in such a breeding strategy. However, up until now, no serum proteins have been associated with cattle resistance to *M. bovis* infection. The present study aimed to investigate the differential expression in serum protein profiles between bTB-resistant and bTB-susceptible Mexican dairy Holstein-Friesian cattle following long-time *M. bovis* exposure (>6 years). Primary amine oxidase (AO), Complement component 5 (C5), and serotransferrin (TF), were found overexpressed in serum of bTB-resistant cattle compared to bTB-susceptible cattle. We then developed enzyme-linked immunosorbent assays (ELISAs) for each of these novel biomarkers and obtained an overall sensitivity higher than 77% and a specificity of at least 83% in field diagnosis. Our results provide potential novel targets for breeding purposes to improve the resistance of cattle to bTB and contribute to the success of eradication campaigns.

## Materials and Methods

### Cattle Population

The present study included Holstein-Friesan dairy cows, 6–8-year-old, born and raised in eight -different dairy farms located in Jalisco, Mexico, a geographic area with known prevalence and active transmission of *M. bovis* (40). Individuals were selected from herds with naturally *M. bovis*-infected animals confirmed by *M. bovis* positive culture, detected during on-going national bTB surveillance and eradication campaign of the Mexican National Service of Agro-alimentary Health, Safety, and Quality (SENASICA). Those herds were at active episodes of infection and were quarantined at the control campaign. All animals were tuberculin tested [single caudal fold (intradermal) test—CFT or intradermal comparative cervical tuberculin test] at intervals of 60–90 days, with restriction from trading and permanent identification and subsequent culling of reactors. The prevalence of bTB within those dairy farms was greater than 30% (some herds with a prevalence of up to 60%). All herds had similar husbandry conditions: a semi-intensive system, housing in barns and grasslands after milking, twice a day, fed with forage, grains, and supplemented during the lactating period. The size of herds was 70–180 milking cows (median of 90) with average milk production of 53,795 kg.

### Phenotype and Exposure Definition

Infected cows (susceptible) were animals that had a positive CFT result (bTB reactors), and all these animals were also positive for the whole blood IFN-γ (interferon-gamma) release assay (IGRA, BOVIGAM Prionics, Thermo Fisher Scientific). When the IGRA is used in parallel with the CFT, the sensitivity for detecting *M. bovis* infected animals increases (41). Furthermore, all these animals were confirmed for bTB by detailed postmortem examination of visible lesions and *M. bovis* culture. They all had gross visible lesions in lymph nodes (head, thorax, and abdomen) or lungs and *M. bovis* positive culture. Lesions were processed for *M. bovis* culture on Lowenstein-Jensen and Stonebrink media at 37°C for 12 weeks. This conservative classification is consistent with the current formal definition of a potential bTB case of SENASICA from Mexico and the Animal and Plant Health Agency (APHA) from the UK. Cows with positive results for CFT or IGRA with no visible lesions and negative *M. bovis* culture were excluded from the study. Not infected animals (resistant) were cows that resulted negative for CFT and negative for IGRA (nonreactors). In addition, all these cattle were confirmed bTB-negative by detailed postmortem examination (i.e., absence of lesions and negative *M. bovis* culture) to avoid inclusion of *M. bovis*-infected but unresponsive (anergic) animals (41, 42).

According to Ring et al. (27), all the cattle included in the present study had a potential bTB exposure: cows within a herd were deemed exposed to bTB if (1) any herd-mate was identified with a lesion at slaughter after being removed as a bTB reactor, (2) any herd-mate was identified as bTB-infected by detailed postmortem examination, or (3) two or more herd-mates were removed from the herd as bTB reactors (27). Transmission of *M. bovis* infection from a reactor to an in-contact animal within a group can occur in a 12-mo period (43). However, tuberculin reactions are early detectable within 42 days following infection, and IFN-γ levels are correlates of infection as early as 14 days after challenge, regardless of the infective dose (41, 44, 45). In the present study, the random selection of cattle took into account infected and not infected in-contacts born and lived in the same herd (close-contacts) and were matched by year of birth: for each not infected cow, one or two infected cows of the same age were included. Cattle moved into herds were excluded from the study. Only animals older than 6 years but younger than 8 years were included in the study. Due to farmers' policies, culling of reactors was carried out only if the cattle were aged 6 years or older. Animals in which the gross pathology and *M. bovis* culture could not be performed were not included in the study. Therefore, we were not able to include younger animals.

Our design sought a long-term period of exposure to the pathogen, intending to reduce the probability of including animals that have yet to be exposed to an infective dose, which can be as minimum as one CFU of *M. bovis* (44), while avoided immunodeficiencies of the elderly. We included contemporary herd-mates potentially exposed to the pathogen with the same probability (same age, husbandry and environment, and lifelong exposure) but with a different outcome (infected or not infected) (46). These binary trait definitions have been used in cross-sectional studies seeking features of bTB resistance in cattle (9–12).

#### Samples

Two hundred thirty-four cows were blood sampled for the biomarkers' discovery study: 96 resistant and 138 susceptible to bTB. Animals were tuberculin tested at farms and blood sampled for sera and IGRA at slaughterhouse pens. Blood samples were taken into Vacutainer tubes (Becton and Dickinson, GDL, MX) with and without anticoagulant (lithium heparin). Samples were immediately protected from heat and sunlight after collection and processed within 2 h after sampling in the laboratory. After clot formation, serum was obtained by 15 mins centrifugation at 12,000 × *g*, 4°C, and stored at −20°C until use.

Although the sample size was not calculated, we had enough experimental power at ensuring accurate phenotype definitions by including not infected animals that have had a high probability of exposure to *M. bovis* and derived from epidemiologically comparable herds (11).

For biomarkers validation, a pilot study was performed individually with in-house ELISAs in another set of 72 serum samples collected from cattle without bTB (*n* = 36) and cattle with bTB (*n* = 36), both diagnosed with or without any other infectious disease (positive to specific RT-PCR tests in clinical samples) or metabolic disease from the same herds described above. Two cases each of the most frequent diseases in the geographical region of the sampled cattle were included per group: (1) *Mycobacterium avium* sp. *paratuberculosis* (MAP)/Johne's disease, (2) *Brucella abortus*, (3) bovine herpesvirus 1 (BoHV-1), (4) bovine viral diarrhea virus (BVDV), (5) bovine parainfluenza virus 3 (BPIV-3), (6) *Leptospira* spp. infection (*L. interrogans* serovar *hardjo* type hardjoprajitno or *L. borgpetersenii* serovar *hardjo* type hardjo-bovis), (7) hypocalcemia, (8) ketosis, and (9) hypomagnesemia. Serum samples from 18 active clinical cases of cattle diagnosed with different infectious or metabolic diseases concomitant to bTB were included as a single group (bTB+IMD). While samples of 18 cattle matching the same diseases without bTB were grouped (bTB-IMD). The comparison included the samples of 18 cattle with bTB without any other clinical disease (bTB+) and 18 samples from cattle resistant to bTB (bTB–), used to standardize ELISAs. Additionally, blood samples of 18 healthy cattle (HS) from a bTB-free, paratuberculosis-free, and brucellosis-free herd (controls; no clinical or laboratory tests and negative to IGRA) were also included.

### A Three-Step Serum Proteome Analysis

#### Highly Abundant Proteins Removal

To reduce individual variation and increase the chance to identify valid biomarkers, all 96 serum samples from resistant cows were pooled. Similarly, serum samples from 138 susceptible cows were pooled. Samples were therefore compared on a one-to-one basis as resistant and susceptible. Proteomic analysis to identify potentially rare biomarkers from serum samples requires depletion of over-abundant proteins and further fractionation (47). To deplete highly abundant proteins (such as albumin), we used precipitation with 10% w/v TCA in cold acetone (48). Four volumes of ice-cold acetone containing 10% w/v TCA were rapidly added to serum pooled samples and immediately mixed by gentle vortexing. The mixtures were incubated at −20°C overnight and centrifuged at 15,000 x *g* for 20 min, 4°C. The precipitate was washed twice with 1 ml of ice-cold acetone on ice for 15 min and centrifuged as above. The precipitated proteins were lyophilized, resuspended in 1 × PBS (120 mM sodium chloride, 1.2 mM sodium phosphate monobasic, 2.8 mM potassium chloride, 8.8 mM sodium phosphate dibasic, pH 7.4), and quantified using the Pierce bicinchoninic acid microplate assay (Thermo Fisher Scientific, CA, USA), following the manufacturer's instructions. For qualitative analysis, 10 μg of protein per well of crude serum, depleted serum (precipitate), and the albumin-rich fraction (supernatant) were resolved by 1.5 mm, 10% SDS-PAGE, carried out at a constant voltage set to 100 V for 1 h, and Coomassie blue stained (Supplementary Figure 1).

#### Serum Proteome Fractionation and Comparative Analysis

**Figure 1:**
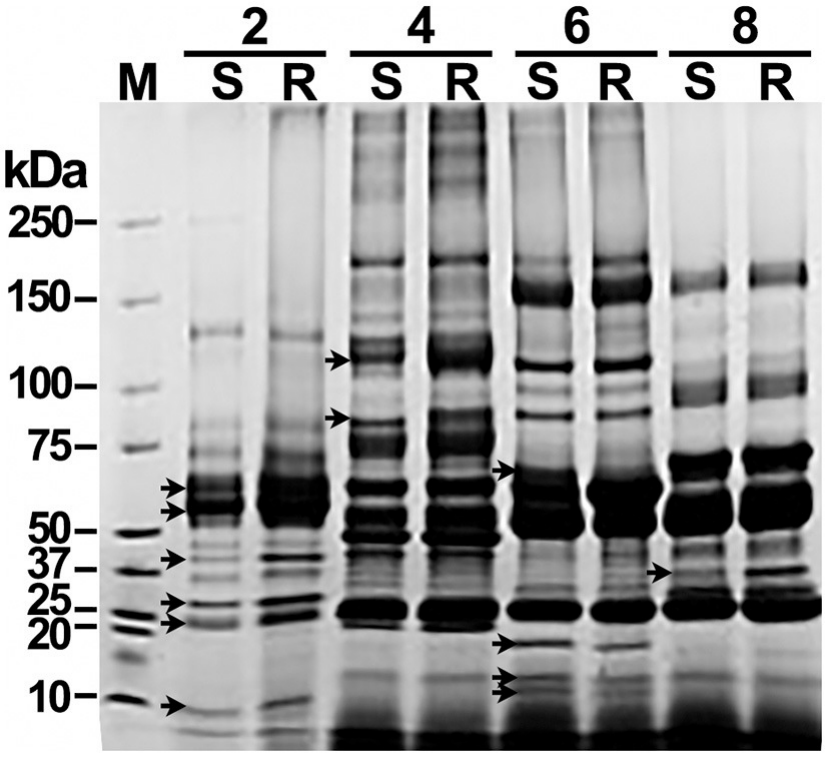
Offgel separation of serum proteins by pI and matching by molecular weight reveal significant differences between susceptible and resistant cattle. Samples of pooled sera depleted of high-abundance proteins were fractionated by pH by offgel electrophoresis. Single offgel fractions were separated on a 4–20% gradient SDS-PAGE, loaded with 10 μg of protein per well by pairing samples from susceptible (S) and resistant (R) cattle. Proteins were Coomasie blue stained. A paired comparative densitometry analysis was carried out to seek differences of ≥ 2 times among pairs of protein bands. A Coomassie blue-stained SDS-PAGE of serum fractions 2, 4, 6, and 8 representatives of that with significant differences in protein bands (arrows) is shown. M, protein molecular weight marker.

Each of the pools of abundant-proteins-depleted sera was fractionated using OFFGEL electrophoresis. The 3100 OFFGEL electrophoresis fractionator and an OFFGEL pH 3–10 kit (Agilent Technologies, CA, USA) were used with a 12-well configuration following the supplier's protocol. The IPG gel strips (13 cm length, pH 3–10) were rehydrated with IPG Strip Rehydration Solution in the assembled device with 40 μl per well for 15 min. Two hundred μg of serum proteins were diluted in protein OFFGEL solution [8 M urea, 2 M thiourea, 40 mM 1,4-dithio-DL-threitol (DTT), and 2% v/v ampholytes pH 3–10] to a final volume of 1.8 ml and 150 μl of sample was loaded into each well. Proteins were submitted to isoelectric focusing (IEF) until reaching 50 kVh with a maximum voltage of 4000 V, 50 μA 200 mW, and a hold setting of 500 V. Twelve individual fractions were recovered and further resolved by molecular weight (kDa) through 1.5 mm, 4–20% gradient SDS-PAGE, under reducing conditions. To facilitate comparisons, fractions were loaded in SDS-PAGE (10 μg/well) organized by pairs matching resistant (R) with susceptible (S). SDS-PAGE was carried out at a constant voltage set to 100 V for 1 h. Gels were stained with Coomassie brilliant blue; images were recorded with a Gel Doc™ XR+ system (Bio-Rad Laboratories, USA) and analyzed by densitometry with Quantity One software (Bio-Rad Laboratories, USA). Single serum fractions that presented differences of two or more times the quantity of a band among paired lanes (Figure 1) were subsequently analyzed by two-dimensional gel electrophoresis (2D-GE).

#### Two-Dimensional Gel Electrophoresis

Individual sera fractions 2, 4, 6, 8, and 10 were respectively pooled (R or S) and processed to remove OFFGEL solution components (through HiTrap desalting columns) before 2D-GE. Pooled sera fractions containing 150 μg of protein each was diluted in a final volume of 350 μl of IPG rehydration buffer (8 M urea, 2 M thiourea, 4% w/v CHAPS, 0.2% DTT, 0.5% IPG buffer, 0.2% w/v Bio-Lyte 3/10 ampholytes, and 0.002% bromophenol blue). The IPG gel strips 7 cm, pH 3-10 (ReadyStrip, Bio-Rad Laboratories, USA) were rehydrated with this solution for 20 h using a DryStrip reswelling tray (GE Healthcare) covered with 1.6 ml of mineral oil. After rehydration, IEF was carried out on an IPGphor (Amersham Biosciences) to reach 52 kVh. Following IEF, gel strips were equilibrated in equilibration buffer (6 M urea, 50 mM Tris-HCl, pH 8.8, 30% v/v glycerol, 2% w/v SDS and 1% DTT) for 15 min. The strips were further transferred to a buffer containing 2.5% iodoacetamide for 15 min. After equilibration, the second dimension was performed on 1.5 mm, 12% non-gradient polyacrylamide gels in a Mini-Protean Tetra Cell chamber (Bio-Rad Laboratories, USA). SDS-PAGE was carried out at a constant voltage set to 120 V for 1.5 h. After protein fixation for 1 h with 10% methanol and 7% acetic acid, the gel was stained using colloidal Coomassie Blue (G-250, Bio-Rad Laboratories, USA). Gel images were taken with a CCD high-resolution camera using the Gel Doc™ XR+ system (Bio-Rad Laboratories, USA). To increase confidence in the results obtained from the 2D gel electrophoresis, gels were run in triplicates with samples from each pooled fractionated sera from R and S cattle, respectively, for quantitative analysis. Gel image analysis included image alignment, spot detection, background subtraction, spot measurement, and spot matching using PDQuest 2-D analysis software (Bio-Rad Laboratories, USA). Coomassie Blue-stained spots were quantified based on their relative volumes (the spot volume divided by the total volume over the whole set of gel spots, according to the instructions provided by the manufacturer). Protein profiles were matched within each R and S gels, and then relative volumes of matched spots were compared between R and S samples for statistical analysis using the Student's *t*-test (*p* < 0.05). A factor greater than a 1.6-fold increase in the spot's average density in one group compared to the other was reported as overexpression.

### In-Gel Digestion and Mass Spectrometric Analysis

Differentially expressed protein bands were excised from the Coomassie-stained gels, distained, and digested with sequencing-grade trypsin (Promega, Madison, WI, USA). Nanoscale liquid chromatography separation of tryptic peptides was performed with a nanoAcquity ultra-performance liquid chromatography (UPLC) system (Waters Corp., MA, USA), equipped with a Symmetry C18 Trap Column (5 μm, 20 mm ×180 μm, 100 A, 2G, Waters) and a PST BEH C18 (1.7 μm, 100 mm × 75 μm, 130 A, 10K psi, Waters) analytical column. The lock mass compound human [Glu1]-Fibrinopeptide B (Sigma-Aldrich) (load mass 1 of 571.6852 m/z, and load mass 2 of 785.8426 m/z) was delivered at 0.5 μL/min at a concentration of 200 fmol/ml to the mass spectrometer. Mass spectrometric analysis (LC-MS/MS) was carried out in a Synapt-HDMS Q-TOF (Waters). The spectrometer was operated in V-mode, and analyses were performed in positive mode ESI. The acquisition window in MS mode was 400–2000 (m/z), and an MS/MS mode was 50–2000 (m/z). The charges of acquired ions were 2+, 3+, and 4+. The data acquisition was set at Data Dependent Acquisition (DDA). MS and MS/MS spectra were acquired at a fragmentation energy ramp of Low Mass Collision Energy (LM CE) of 15–45 volts and High Mass Collision Energy (HM CE) of 15–55 Volts. The Lock Mass reference sprayer was sampled every 30 s.

### Data Analysis and Protein Identification

MS/MS spectra data sets were used to generate PKL files using Protein Lynx Global Server v2.4 (PLGS, Waters). Proteins were then identified using the Mascot search engine algorithm (Matrix Science, London; http://www.matrixscience.com). Searches were conducted using the NCBIprot (128,624,863 sequences), the *Bos taurus* protein database (UP9136; 37,880 sequences), and the contaminants database (262 sequences). Trypsin was used as the specific protease, while one missed cleavage was allowed with a 0.6 Da tolerance set for the precursor and the fragment ion masses, while carbamidomethyl (C) and oxidation (M) were selected as variable modifications. Proteins were identified based on a minimum of two unmodified highly scoring unique peptides per protein at a false discovery rate (FDR) of 1%. Proteins identified were: (1) amine oxidase (EC 1.4.3.21), (2) complement component 5 (C5), (3) serotransferrin and (4) haptoglobin (Table 1). The former three were over-expressed in R cattle sera while the latter in sera of S cattle. Only overexpressed proteins in the resistant group were assessed by immunodiagnostic tests (ELISAs).

**Table 1:** Identification of serum proteins differentially expressed in bTB-resistant/bTB-susceptible cattle by 2D-GE.

**Spots no.^a^**	**Average ratio of abundance**	***P* value^b^**	**Peptides matched/sequence coverage**	**Mascot score^c^**	**Protein name**	**Accession entry^d^**	**Theoretical mass (kDa)/pI**
1	1.6	<0.0001	5/4%	87	Complement component 5	A0A0F6QMJ3/ F1MY85	188.677/6.20
2	8.5	<0.0001	8/12%	209	Amine oxidase	E1BJN3	76.896/5.69
3	2.1	<0.0001	17/24%	250	Serotransferrin	Q29443	77.689/6.75
4	−1.8	<0.0001	4/12%	124	Haptoglobin	G3X6K8	41.954/7.10

a *Numbers correspond to spots circled in Figure 2*.

b *Student's t-test*.

c *Individual ions scores > 35 indicate identity or extensive homology (p < 0.05)*.

d *UniProtKB*.

### ELISAs

#### Production of Rabbit Polyclonal Antibodies Against Candidate Biomarkers

Hyperimmune antisera were produced from 2 female New Zealand white rabbits for each protein. Each pair of rabbits was injected subcutaneously at multiple sites with 50 μg antigen (0.5 mg/ml PBS) emulsified in an equal volume of TiterMax Gold adjuvant (T2684, Sigma-Aldrich, MO, USA); rabbits were inoculated thrice at 2 weeks intervals. Antigens for injection were obtained as follows: complement C5 was isolated from bovine serum as previously described (49); bovine plasma amine oxidase (M4636) and bovine serum transferrin (T1428) were obtained from Sigma-Aldrich (Sigma-Aldrich, MO, USA). Serum from each pair of rabbits was collected 2 weeks after the last immunization by cardiac puncture under terminal anesthesia and pooled. Immunoglobulins were precipitated with 50% ammonium sulfate pH 6.8, followed by dialysis against 10 mM PBS pH 7.6 (100 × v/v) and chromatography purification on DEAE Sepharose equilibrated with the same buffer (50, 51). Rabbit IgGs were quantified by UV absorbance at 280 nm and adjusted to 1 mg/ml, based on a calculated extinction coefficient of O.D._280_ = 1.0 ≈ 1.4 mg/ml IgG. Purity was analyzed by 12.5% SDS-PAGE and Coomassie blue staining and immunoblotting (Supplementary Figure 2). Detection antibodies were obtained by conjugating purified rabbit IgGs with HRP (P8125, Sigma-Aldrich, MO, USA) by the periodate method (52). The IgG-HRP products were dialyzed overnight against 1X PBS at 4°C. Capture and detection (HRP-conjugated) rabbit antibodies were preserved with BioStab (55514, Sigma-Aldrich, MO, USA) and used to implement indirect sandwich ELISAs. The purified proteins used to generate the antibodies were also used as standards.

#### Performance Evaluation

Capture and detection antibodies were titrated in a range of standard concentrations as well as protein standards (capture antibodies: 4, 2, 1 and 0.5 μg/ml; detection antibodies: 0.4, 0.2, 0.1, 0.05 and 0.025 μg/ml; protein standards for TF 10, 5, and 1 mg/ml and for AO and C5 100, 10, 1, and 0.1 ng/ml). All parameters were analyzed in triplicate. Other dose-response curves for the concentration of each standard in the sample matrix (bovine serum: 0, 10, 25, 50, and 100%) were performed. Two standard dilution buffers were assayed: 10% blocking buffer with 0.05% Tween 20 in 1X PBS; and 2% (v/v) horse serum (H1270, Sigma-Aldrich, MO, USA) 0.05% Tween 20 in 1X PBS. Three blocking buffers were evaluated: 1% casein in 1X PBS (1610783, Bio-Rad Laboratories, USA); 0.5% enzyme immunoassay-grade fish skin gelatin (G7041, Sigma-Aldrich, MO, USA) in 1X TBS (20 mM Tris and 150 mM NaCl, pH 7.6); and 5% nonfat dry milk (1706404, Bio-Rad Laboratories, USA) in 1X TBS. The wash buffer was 0.05% Tween 20 in 1X PBS. The HRP substrate was 1% tetramethylbenzidine (TMB) dissolved in Dimethylsulfoxide (DMSO), diluted 1:100 in 0.1 M sodium acetate (pH 6.0) and 0.005% hydrogen peroxide (added just before using the substrate). The stopping solution was 1.5 N sulfuric acid. ELISAs were performed in 96-well clear flat bottom polystyrene High Binding microplates (Costar 9018, Corning, MA, USA). The optimal concentration for each antibody pair was determined by the lowest signal-to-noise ratio of the mid and low standards compared to a top standard. The signal-to-noise ratio was obtained by dividing the average optical density (OD) of each standard by the average OD of its corresponding zero standards. The optimal blocking buffer was 0.5% fish skin gelatin, which produced the lowest OD reading difference between 0, 10, and 50% of the sample matrix plus standards for the entire tested interval.

#### Lowest Limit of Detection, Precision, Linear Range, and Recovery Rate

The precision of the assay was determined through the construction, modeling, and evaluation of the concentration curve of protein standards using all the combinations of reagents described above. Each detection was repeated ten times for three different days by two independent people. The four-parameter logistic (4PL) fit was performed as the reference model for the standard curves (53). The linear range of the effective concentration range of each candidate biomarker that each ELISA can detect was determined with eight serially diluted concentrations of each protein performed in triplicate. The linear range was confirmed when the regression coefficient *R* ≥ 0.90 and the minimum of the linear range were higher than the LLOD. The LLOD was calculated by the mean plus two standard deviations (M+2SD) of the diluent OD measured 20 times.

The recovery rate was used to evaluate the accuracy of the ELISA tests. A sample of high-concentration standard (A) was added to a sample of low-concentration standard (B) with a volume ratio (A: B) ≤ 1:9. The recovery rate R was calculated as follows, *R* = *C*×(*V*0+*V*)−(*C*0×*V*0)/(*V*×*CS*)×100%, where V is the volume of sample A, V0 is the volume of sample B, C is the concentration of the mixture of A and B, C0 is the concentration of sample B, and CS is the concentration of sample A.

### Validation Analysis

The concentration of AO, C5, and TF in cattle sera was measured using in-house-built and standardized indirect sandwich ELISAs. Equal numbers of serum samples (n=18 in each group) from cattle with or without bTB identified by skin test, IFN-gamma test, lesions, and *M. bovis* culture, with or without another infectious or metabolic disease, plus control healthy cattle, were included for the analysis. Flat bottom 96-well plates were coated overnight at 4°C with 50 μl of 2 μg/ml polyclonal rabbit capture antibodies against bovine AO, C5, or TF diluted in 50 mM sodium carbonate, pH 9.6. The ELISA components and serum samples were equilibrated at room temperature (RT) for 30 min before use. The detection procedure was as follows. Coated plates were washed with 0.05% Tween 20 in PBS, three times (250 μl/wash/well, 15-s soaks between washes; each washing step) using an automatic plate washer (1575, Bio-Rad Laboratories, USA). Blocking buffer (150 μl/well, 0.5% fish skin gelatin in TBS) was immediately added and incubated for 1 h, at RT shaking on an orbital shaker (2314Q, Thermo Scientific, CA, USA). After washing, 100 μl of standard sample dilutions and serum samples diluted 1:10, both in 0.05% gelatin-0.05% Tween 20 in 1X TBS, were added in triplicates. The plates were sealed with film and incubated for 2 h at RT, shaking. The plates were then washed, and 100 μl of 0.1 μg/ml HRP-conjugated detection antibodies diluted in 0.05% gelatin-0.05% Tween 20 in 1X TBS were added to wells. The sealed plates were left shaking for 1 h at RT, after which wells were washed once more. TMB substrate solution was added to each well (100 μl) and incubated in the dark without shaking for 15 min at RT. Finally, 100 μl of 1.5 N sulfuric acid was added per well, and the plate was gently mixed by hand. Plates were scanned with the iMark plate reader (Bio-Rad Laboratories, USA) at 450 and 595 nm, and the delta OD (OD450–OD595) was used as the OD data point.

The standard curves were provided from 7 dilutions of each purified standard and zero (AO: 400, 200, 100, 50.0, 25.0, 12.5, 6.25 and 0 ng/ml; C5: 300, 150, 75, 37.5, 18.75, 9.38, 4.69 and 0 ng/ml and TF: 8.67, 5.78, 3.85, 2.57, 1.71, 1.14, 0.57 and 0 mg/ml). The cut-off values were chosen by a percentile method at which the highest sensitivity and specificity are reached and ROC (receiver operating characteristic) evaluating the discriminative capacity of the diagnostic tests. The detection effect was evaluated according to the area under the ROC curve (AUC-ROC).

### Statistical Analysis

Comparisons between groups were conducted using the Student's *t*-test (resistant vs. susceptible) in the abundance of protein spots in 2D-GE determined by PDQuest 2-D analysis software and concentration of candidate biomarkers in individual serum samples quantified by the in-house ELISAs. Assessment of specificity, sensitivity, and area under the curve (AUC) was carried out using Receiver Operating Characteristic (ROC) curves. Kruskal-Wallis nonparametric test with Dunn's multiple comparison *post hoc* test was carried out to compare the mean rank serum levels of candidate biomarkers in each group of cattle (bTB+, bTB+IMD, bTB-IMD, HS) against the mean rank serum levels of the bTB-resistant cattle (bTB-). Nonparametric tests were selected after data were subjected to the D'Agostino-Pearson omnibus K2 normality test that showed that the data were not normally distributed. All statistical analyses were performed with Prism 6.0 (GraphPad Software Inc., CA, USA). *p* < 0.05 was statistically significant.

## Results

### Identification of Potential Biomarkers

To enhance the resolution of the comparative analysis, depleted sera were further fractionated by IEF with OFFGEL electrophoresis. Individual OFFGEL fractions of R and S sera were further paired and resolved by one-dimension SDS-PAGE. Afterward, densitometry of bands was performed with Quantity One software (Bio-Rad Laboratories). Protein patterns showing significant differences amongst bands of R and S fractionated sera are shown in Figure 1 (indicated with arrows). OFFGEL fractions 2, 4, 6, 8, and 10 showed a ≥ 2-fold difference in density of some protein bands. Those serum fractions were subsequently combined by R or S origin and resolved by 2D-GE in triplicates for accurate excision and protein identification. OFFGEL fractions 1, 3, 5, 7, 9, and 11 did not show significant differences, and fraction 12 had not defined protein bands (not shown), and thus all they were excluded from further analysis.

Representative images of 2D-GE of processed sera from R and S cattle are presented in Figure 2. Potential differences of abundance in protein spots were evaluated using PDQuest 2-D software (Bio-Rad Laboratories). The software detected 173 matched spots common to the two groups of cattle. Four spots were consistently differentially expressed between groups (*p* < 0.0001) (Figure 2). Three protein spots (encircled and numbered 1, 2, 3 in 2D-GE) were differentially upregulated in bTB-resistant cows compared to cows with bTB (≥ 1.6). In contrast, one protein spot (number 4) was upregulated in cattle with bTB compared to cattle resistant to bTB (> 1.8). Proteins identified overexpressed were excised from gels, in-gel digested with trypsin, and processed for peptide mass-spectrometric identification by LC/ESI-MS/MS. Results are shown in Table 1. Two of these protein spots were identified as the same protein (number 3) with different pI. Proteins overexpressed in bTB-resistant cattle were AO, C5, and TF, while the upregulated protein in cattle with bTB was haptoglobin. Protein identification was unambiguous in all cases as judged by peptide mass accuracy and sequence coverage (Supplementary Table 1).

**Figure 2:**
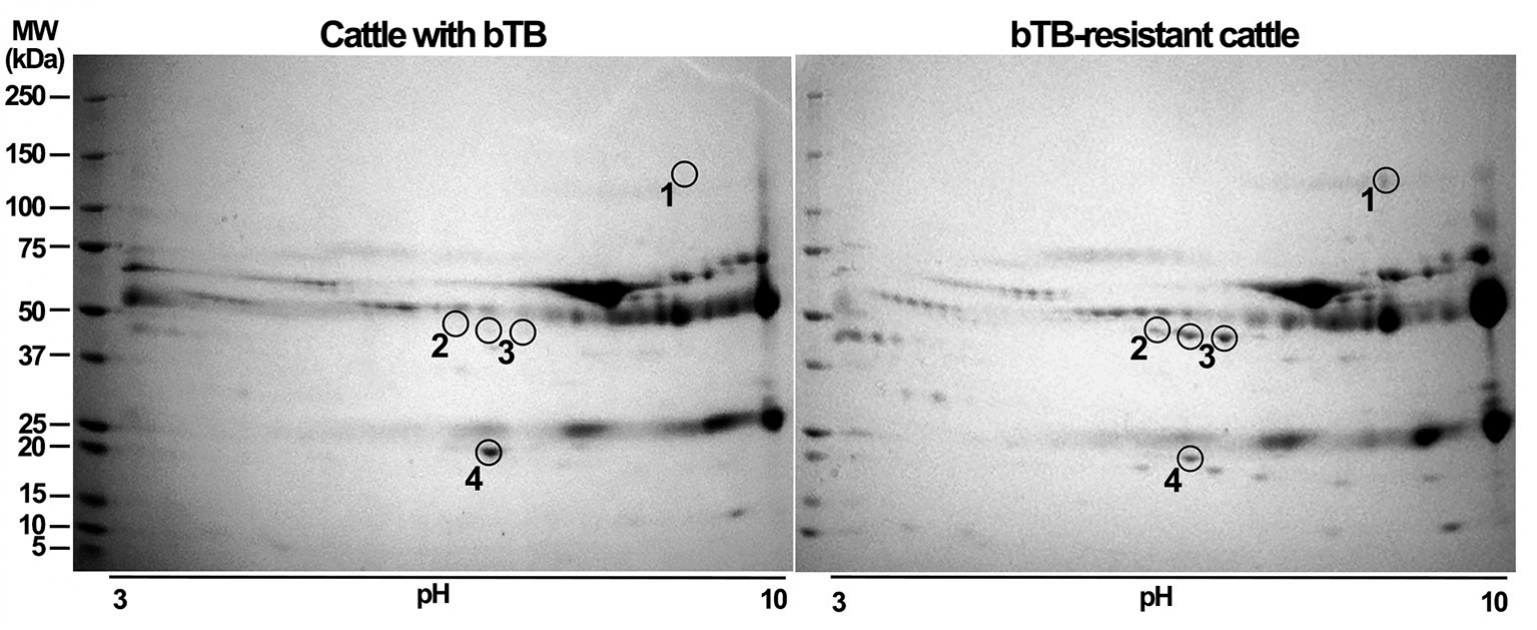
Resolving fractions of sera by two-dimensional gel electrophoresis (2D-GE) enhances detection and precise excision of overexpressed proteins of susceptible and resistant cattle. Offgel fractions 2, 4, 6, 8, and 10 of sera from bTB-susceptible and bTB-resistant cattle were independently pooled, cleaned, separated by 2D-GE and stained with Coomassie blue. A comparative analysis of protein amount was implemented using PDQuest 2-D software. Proteins with significant differences (*p* < 0.05, Student's t-test) were excised and processed for LC/ESI-MS/MS analysis. We show a representative two-dimensional gel of proteins from bTB-susceptible cattle and one of that of bTB-resistant cattle. Proteins differentially expressed among gels are enclosed in circles and numbered. Encircled protein spots close to number 3 correspond to the same protein, but with a different isoelectric point. Protein molecular weight markers (M) were ran along the samples to determine the relative molecular weight of proteins.

### Development of ELISAs for the Evaluation of Potential Biomarkers

Before evaluating AO, C5, and TF, as potential biomarkers of resistance to bTB, there was a need to develop an assay that would allow the evaluation for the presence of these proteins in cattle samples. Accordingly, we developed and standardized capture and detection rabbit antibodies and individual ELISA tests to determine the concentration of these proteins in serum samples derived from 18 cattle resistant to bTB and 18 cattle with bTB.

**Figure 3:**
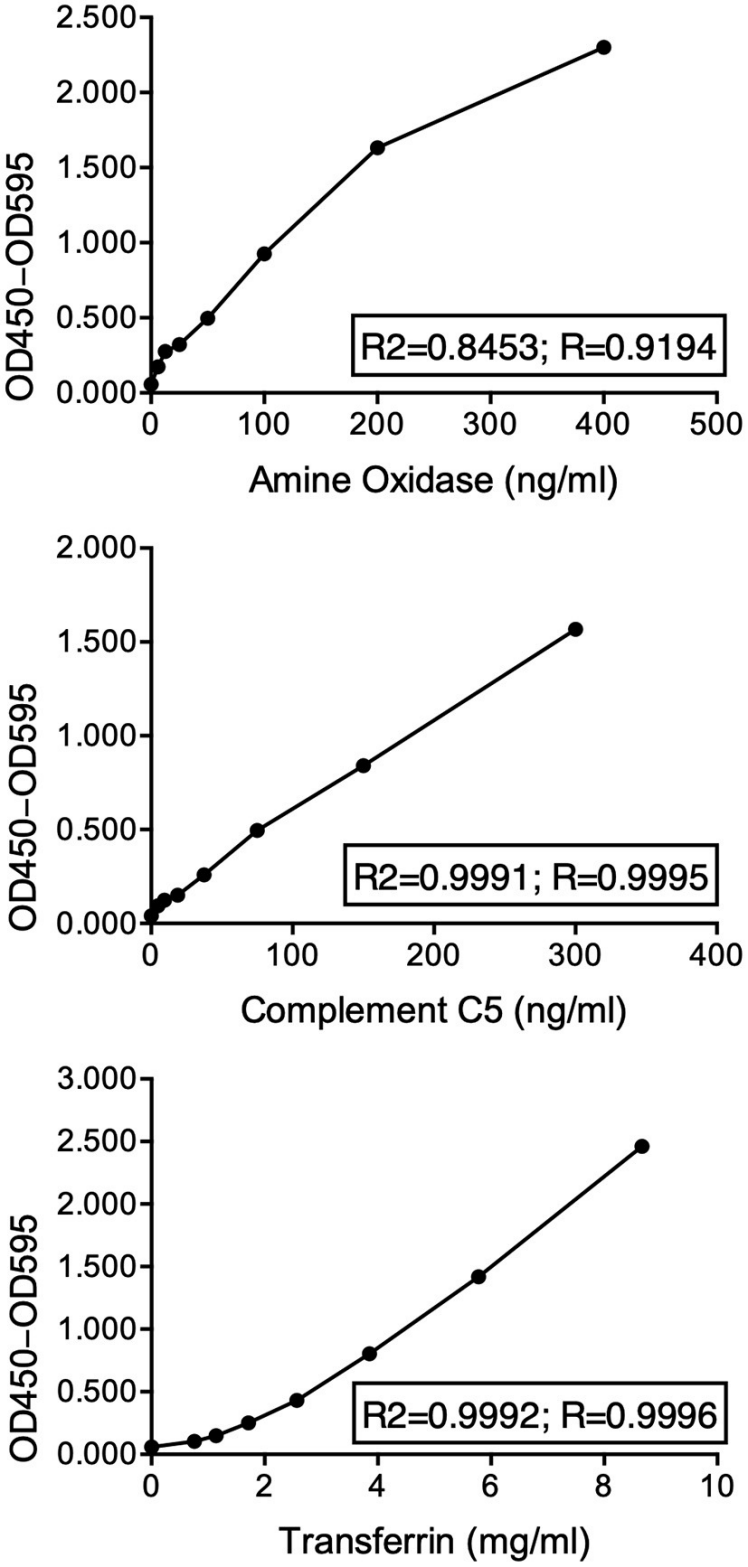
Nonlinear regression of OD and concentration of amine oxidase, complement component 5, and transferrin confirmed precision of each in-house sandwich ELISA in their linear range. Triplicates of eight serially diluted concentrations of each purified protein were quantified by their respective in-house sandwich ELISAs. ODs (OD 450 nm – OD 595 nm) vs. concentrations were modeled by a sigmoidal, four-parameter logistic (4PL) fit regression, and the coefficient of determination (*R*^2^) and the coefficient of correlation (*R*) were estimated.

**Figure 4:**
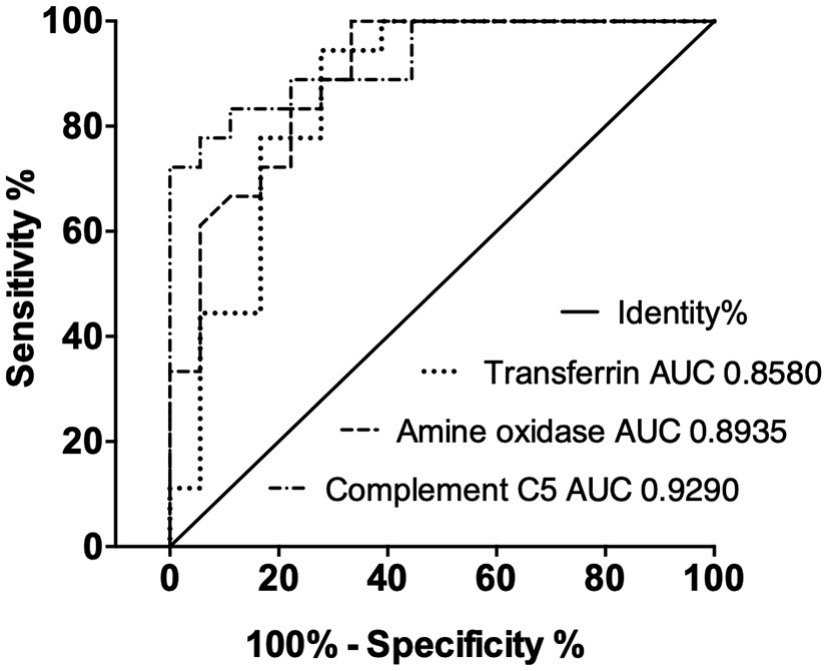
Receiver-operating characteristic (ROC) curves of candidate biomarkers show good correct diagnosis indexes of ELISAs in clinical samples. The areas under the ROC curves (AUCs) are above 85%.

The performance of each ELISA against these novel biomarkers is summarized in Table 2. Overall, the three ELISAs showed a series of acceptable performance parameters: linear ranges of 6.25–400, 4.69–300 ng/ml, and 0.76–8.67 mg/ml, LLOD of 3.13, 2.34 ng/ml, and 0.51 mg/ml, for AO, C5, and TF. The positive detection rate for AO, C5, and TF was 93.8, 94.3, 84.6%, respectively. Linear ranges showed correlation coefficients higher than 0.90, and LLOD could be separated from the background (Figure 3). The areas under the ROC curves (AUC-ROC) of AO, TF, and C5 were 0.8935 (0.7906–0.9964, 95% CI), 0.8580 (0.7291–0.9869, 95% CI), and 0.9290 (0.8484–1.010, 95% CI), respectively (Figure 4). As observed, the mean values of AUC-ROC are greater than 85% in all in-house ELISAs, indicating a very high probability of accurate and true results with these assays. The correct diagnosis index, defined as sensitivity % - (100% – specificity %), reached its maximum at a concentration of 190.0 ng/ml for AO, 2.0 mg/ml for TF, and 176.0 ng/ml for C5. Thus, for AO, TF, and C5, the cut-off values were chosen as 192.0 ng/ml, 2.1 mg/ml, and 176.5 ng/ml. These cut-off values provided the highest sensitivities and specificities for each ELISA. The sensitivities were 72.2, 77.8, 77.8%, and the specificities were 94.4%, 94.4%, and 83.3%, for AO, TF, and C5, respectively (Table 2). In summary, the ELISAs were highly predictive of real results with a high capacity to detect significant differences between cattle with bTB and cattle resistant to bTB.

**Table 2:** Performance parameters of each in-house ELISA.

	**Amine oxidase**	**Complement C5**	**Serotransferrin**
Linear ranges	6.25–400 ng/ml	4.69–300 ng/ml	0.76–8.67 mg/ml
LLOD	3.13 ng/ml	2.34 ng/ml	0.51 mg/ml
Recovery rates	80–120%	80–120%	80–120%
AUC-ROC (95% CI)	0.8935 (0.7906–0.9964)	0.9290 (0.8484–1.010)	0.8580 (0.7291–0.9869)
Cut-off values	192.0 ng/ml	176.5 ng/ml	2.1 mg/ml
Sensitivity %	72.2%	77.8%	77.8%
Specificity %	94.4%	94.4%	83.3%
Odds ratio	13.00	14.00	4.67
Positive predictive value	93.8%	94.3%	84.6%
Negative predictive value	74.3%	78.4%	76.2%

### Evaluation of Biomarkers as Indicators of Resistance or Susceptibility to bTB

**Figure 5:**
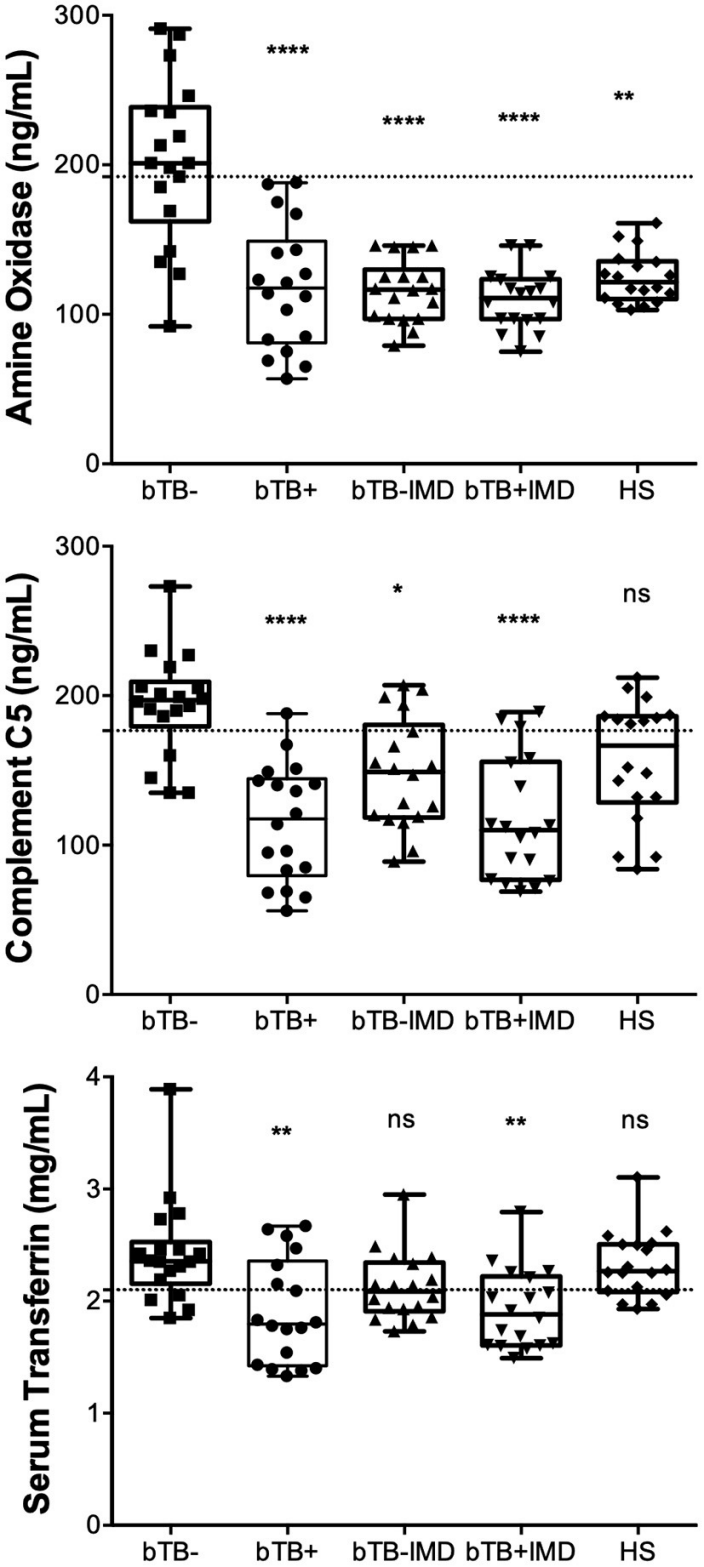
Concentrations of AO, C5, and TF measured in clinical samples by ELISA tests exhibit significant efficacy in diagnosing bTB-resistant cattle. AO, C5, and TF were quantified by the in-house developed and standardized indirect sandwich ELISAs in serum samples (n = 18) of cattle from herds with endemic bTB, but without bTB (bTB-), cattle with bTB (bTB+), cattle without bTB, and with any infectious or metabolic disease (bTB-IMD), cattle with bTB and with IMD, and healthy cattle (HS). Statistical analysis was performed by comparing the mean rank of each group of cattle against the mean rank of the bTB-resistant cattle through the Kruskal-Wallis test. *p* < 0.0001^****^, *p* < 0.001^**^, *p* < 0.01^*^, ns not statistically significant. Gridlines indicate the cut-off values.

In order to validate 2D-GE and LC/ESI-MS/MS data, we performed ELISA analyses for AO, C5, and TF in naturally exposed cattle to *M. bovis*, infected (bTB+) and not infected (bTB-) and with and without a concomitant infectious or metabolic disease (IMD), as well as in healthy cattle (HS) not exposed to *M. bovis*. The mean serum concentration values (± interquartile rank) of AO, TF, and C5, in cattle resistant to bTB and cattle with bTB are summarized in Table 3. Mean serum levels of these novel candidate biomarkers in different groups of cattle are presented in Figure 5. The serum concentration of these three proteins measured in cattle without any concomitant disease was significantly higher in cattle resistant to bTB than in cattle with bTB (≥1.4 times; *p* < 0.001). At the same time, the mean serum concentration of AO was significantly higher in cattle resistant to bTB without any simultaneous disease (bTB-) than in all other groups (*p* < 0.01). C5 mean serum values of the bTB- group were not distinguishable from C5 mean serum values of the healthy group (HS), although they were significantly higher than the rest of the animals in the other groups (*p* < 0.01). TF values in serum of bTB-resistant cattle, with or without an infectious or metabolic disease (IMD), were significantly higher than in sera of cattle with bTB (*p* < 0.001), but like those of HS (*p* > 0.05).

**Table 3:** Concentration values of candidate biomarkers in serum samples of bTB-resistant cattle and bTB-susceptible cattle determined by in-house ELISAs.

**Protein**	**Cattle resistant to bTB (Mean ± interquartile rank, *n* = 18)**	**Cattle with bTB (Mean ± interquartile rank, *n* = 18)**	**Average ratio^a^**	***p* value^b^**
Complement component 5 (ng/ml)	193.8 ± 8.129	114.8 ± 9.225	1.7	0.0001
Amine oxidase (ng/ml)	202.3 ± 13.05	118.6 ± 9.892	1.7	0.0001
Serotransferrin (mg/ml)	2.43 ± 0.108	1.74 ± 0.113	1.4	0.0001

a *bTB-resistant/bTB-susceptible*.

b *Student's t-test*.

## Discussion

In the present study, we have identified that the level of expression of some bovine serum proteins in Holstein-Friesian dairy cows that appear to be resistant to *M. bovis* infection is higher than the expression of the same proteins in cattle that have succumbed to bTB. These serum proteins are AO, C5, and TF and would appear to be promising biomarkers of resistance to bTB.

Many efforts have been directed toward identifying diagnostic protein biomarkers capable of distinguishing between cattle with subclinical *M. bovis* infection (early experimental infection−1 month) compared to cattle with *M. bovis* clinical infection (late experimental infection−4 months) or not infected. Serum levels of fetuin, alpha-1-microglobulin/bikunin precursor protein (AMBP), alpha-1 acid glycoprotein, and alpha-1B glycoprotein were elevated exclusively in *M. bovis* infected animals compared to contemporary controls and *M. paratuberculosis* infected animals. Whereas transthyretin, retinol-binding proteins, and cathelicidin were identified exclusively in *M. paratuberculosis* infection (30). These previous observations suggest that specific mycobacterial infections may evoke particular non-specific responses. However, to our knowledge, none of these proteins correlates with resistance to *M. bovis* infection. Natural resistance to infection in this work is the host's capacity to remain uninfected in spite of prolonged exposure to *M. bovis* challenge (8, 54).

While many efforts seeking to distinguish between cattle resistant and susceptible to *M. bovis* have been made, those efforts have focused mainly on the use of nucleic acid analysis, including single nucleotide polymorphisms (10, 22–25). To the best of our knowledge, this is the first study seeking to characterize serum proteins that may distinguish cattle that are resistant from those which are susceptible to bTB. The characterization of such biomarker proteins provided the opportunity to develop an ELISA test that could be used as a pen test to determine whether cattle are susceptible or resistant to bTB. We evaluated the performance of this test by correctly identifying samples from cattle known to be susceptible to bTB (i.e., showed a sign consistent with bTB infection as described in materials and methods) from those known to be resistant to bTB (i.e., animals that were long term close contacts of animals that were infected with *M. bovis* as described in materials and methods). Our ELISA systems showed linear range of 6.25–400, 4.69–300 ng/ml, and 0.76–8.67 mg/ml, LLOD of 3.13, 2.34 ng/ml, and 0.51 mg/ml, as well as sensitivities of 72.2, 77.8, and 77.8% and specificities of 94.4, 94.4, and 83.3%, respectively, for AO, C5, and TF. Linear ranges showed correlation coefficients higher than 0.90, and LLOD could be separated from the background. The positive detection rate for AO, C5, and TF was 93.8, 94.3, 84.6%, respectively, showing the high sensitivity of these detection systems.

To determine the extent to which these proteins may vary in their concentration in the plasma due to the presence of other infectious diseases, we measured AO, C5, and TF in individual serum samples of cattle exposed to *M. bovis* with a negative or positive diagnosis of bTB that were positive or negative to any unrelated infectious or metabolic disease. In addition, samples from healthy cattle without exposure to *M. bovis* (from a bTB-free herd) were included as controls. Overall, the OD values in our ELISA test of AO, TF, and C5 were significantly higher in cattle resistant to bTB than in those with bTB (≥1.4x, ≥1.7x, ≥1.7x, respectively), regardless they were suffering or not from other unrelated infectious or metabolic diseases (*p* < 0.001). However, unlike TF levels, AO and C5 serum levels were depressed in cattle resistant to bTB with a simultaneous disease (bTB-IMD). Our observations suggest that AO, C5, and TF are reliable markers of a protective host response against bTB and that AO and C5 levels are negatively affected by other pathologies present in cattle resistant to *M. bovis*, like *M. paratuberculosis* infection, and other unrelated bacterial and viral infections or metabolic diseases. Seth et al. (30) reported that the high levels of apolipoproteins in sera of contemporary unexposed controls were depressed in sera of animals after 10 months of infection with *M. paratuberculosis*. Gao et al. (31) reported that the high levels of serum amyloid A and alpha-1-acid glycoprotein in sera of uninfected controls decreased significantly in *M. bovis*-infected cattle. They also observed that serum levels of TF and C-Reactive protein (CRP), an activator of the classical complement pathway, had no significant differences between *M. bovis*-infected cattle and uninfected controls. However, CRP levels in the PPD-B-stimulated blood of *M. bovis*-infected cattle were significantly higher than those in uninfected controls, while TF levels were lower than the unstimulated blood. These authors suggested that PPD-B may activate acute-phase proteins, increasing CRP and decreasing TF (31).

Interestingly, in our study, C5 and TF serum values were similar (*p* > 0.05) between cattle resistant to bTB (bTB-) and the healthy group (HS), while AO levels in sera of these healthy animals were significantly lower compared to those of the bTB-group. Using the cutoff values of our ELISA tests for C5 and TF, more than half of HS animals would be resistant, despite not having been exposed to *M. bovis*. On the opposite, using the AO cutoff values, all HS animals would be susceptible. It is hard to interpret the variation in AO, C5, and TF levels in these not exposed cattle. Although HS cattle were randomly selected from bTB-free, paratuberculosis-free, and brucellosis-free dairy herd, the possibility of exposure to other viruses or bacteria, like environmental mycobacteria, may lead to these variations in the levels of AO, C5, and TF, cannot be excluded. As discussed above, non-specific responses may be related to particular mycobacterial infections or other pathogens. Previous studies have shown that the levels of specific serum proteins are dynamic along with the *M. bovis* infection (30). Our results suggest that the combinational use of these potential biomarkers rather than alone may better determine the resistance phenotype in cattle. Whether the level of these proteins are only markers of the animal status (resistance to *M. bovis* infection) or are indicative of the intensity of the effector response, those are singularly related to the protective response against *M. bovis* infection, and their effect on bTB requires further investigation.

Bovine plasma amine oxidase (AO), also known as bovine serum amine oxidase (BSAO), is a liver-expressed Cu^2+^ containing enzyme, member of the class of copper-containing amine oxidases (AOC) (55). Plasma AOs are considered key enzymes in cell growth and differentiation processes. AO occurs circulating in plasma apparently due to the proteolytic cleavage of the membrane-bound form (55). The highly structural similarities between bovine AO (E1BJN3) and bovine primary amine oxidase—liver isozyme (AOCX, Q29437; 90.81% identity), bovine primary amine oxidase—lung isozyme (O46406; 82.1% identity), and bovine membrane primary amine oxidase, copper containing 3 (AOC3, Q9TTK6; 80.3% identity), strongly support this theory. Indeed, the copper amine oxidase gene encoding BSAO expressed in the bovine liver is different from, but closely related to, the copper amine oxidase gene expressed in the bovine lung, kidney, spleen, and heart (56). AO preferentially catalyzes the oxidative deamination of primary amines; thus, it is referred to as primary-amine oxidase (EC 1.4.3.21) (57). BSAO also catabolizes polyamines (putrescine, spermidine, and spermine) to produce hydrogen peroxide, aldehyde, acrolein, and ammonia (58). These, in turn, can act as antimicrobial agents and signaling molecules that contribute to leukocyte adhesion and cytotoxicity of drug-resistant cancer cell lines (58). Oxidative degradation of polyamines by bovine AO might restore immune response by avoiding M2 polarization and enhancing inducible nitric oxide synthase (iNOS), NO production, and NO-mediated bacterial killing in activated macrophages (59); however, further studies need to be addressed. AOC3 is also known as vascular adhesion protein-1 (VAP-1), a membrane-bound protein that mediates the slow-rolling and adhesion of lymphocytes to endothelial cells (60). AOC3/VAP-1 also contributes to the extravasation of neutrophils, macrophages, and lymphocytes to sites of inflammation and transiently contributes to the antigen-specific CD4+ T-cell traffic to secondary lymphatic tissues (61). AOC3/VAP-1 participates in developing pulmonary inflammation and fibrosis by regulating the accumulation of pathogenic leukocyte subtypes (62, 63). However, the dispersal of effector CD4+ T cells to lung parenchyma or airway mucosa is AOC3/VAP-1 independent (61). Bovine AOC3 has been identified as being upregulated at least two-fold in MAP-immunoreacting cows (64). However, its role in bTB is yet to be determined.

The complement system is a complex enzymatic cascade (consisting of more than 30 proteins in blood plasma) that functions in stepwise activation of several proteases produced by the liver, adipose tissue, leukocytes, and vascular cells (65). C5a and C5b products generated upon the cleavage of C5 by C3/C5 convertases have essential biological activities. C5b, the larger cleavage product of C5, initiates the formation of the cytolytic complex (C5b-9) that causes lysis of bacteria and pathogens, whereas C5a, the smaller product, is a strong chemotactic and spasmogenic anaphylatoxin that mediates inflammatory responses by stimulating neutrophils and phagocytes to the site of injury or infection (66). Downregulation of the complement factor C5 has been detected in dairy cows over-conditioned around calving during the transition period and in dairy cows exposed to heat stress, suggesting that immune function is impaired in these cattle (65, 67). To the best of our knowledge, evaluations of the protein expression of the complement component C5 of dairy cows with bTB are mainly unexplored. The role of complement factor C5 and its association with *M. bovis* infection in dairy cows represents an attractive possibility to be investigated in the future.

Bovine TF is a single-chain ß-globulin that is important for iron (Fe) transport in blood plasma and is mainly produced by hepatocytes, macrophages, and other cells (68). Intraphagosomal *M. tuberculosis* can acquire iron from both extracellular TF and endogenous macrophage sources, except that iron acquisition from macrophage cytoplasmic iron pools may be critical for the intracellular growth *of M. tuberculosis* (69). TF in the circulating plasma and leukocytes play essential roles in reducing iron availability to the pathogen by their high affinity for Fe3+ (70). Iron requires to be complexed to TF for delivery into cells; iron is known to play a role in the immune response to pathogens. It is known that low intracellular iron availability enhances iNOS transcription; Nramp1 can enhance NO formation and other pro-inflammatory immune pathways via modulation of iron homeostasis (71). Such is supported by published data showing a decrease in the TF serum levels of patients with active TB compared to patients with latent TB or healthy controls (72). However, some studies have indicated that overexpression of TF could be a specific biomarker for the diagnosis of Johne's disease (64). Plasma TF concentrations likely vary in cows with Johne's disease depending on the stage of MAP infection, and elevated TF levels may be compensating for impaired iron uptake across the damaged intestinal epithelium in chronic paratuberculosis (64).

In ruminants, serotransferrin (TF) is typically classified as negative acute-phase protein (APP) during acute infections and acute metabolic diseases (73, 74). Chronic infectious diseases of cattle, such as Johne's disease and bovine viral diarrhea, and acute infections by *Haemophilus somnus* or *Trypanosoma vivax*, were characterized by the relatively low TF serum levels in affected individuals compared to healthy controls (68, 75, 76). In contrast, administration of endotoxin, or during ketosis, did not induce changes in TF concentrations (68). In agreement with our study, levels of TF decreased significantly in bovines susceptible to ticks (Holstein, *Bos taurus taurus*) during heavier infestations of *Rhipicephalus microplus*. In contrast, there was no significant decrease in the serum concentration of TF in tick-resistant cattle (Nelore, *B. taurus indicus*) compared to susceptible cattle (77). Low basal levels of TF in tick-resistant animals may reflect that less free iron is available in them than tick-susceptible animals (77). Increased levels of TF in serum of active human TB cases compared to healthy controls appears to be a compensatory mechanism influenced by nutritional deficiencies (78). TF overexpression in human TB and other infectious diseases is more likely linked to protective immune responses, while its downregulation is more often related to immune deficiencies (79). Blood plasma TF levels were higher in patients with drug treatable HIV than patients with drug-resistant HIV; this also correlated with virological status and immune parameters such as CD4 counts (80).

In conclusion, we have identified three significantly overexpressed serum proteins which could be used as potential biomarkers to determine resistance to bTB in diary Holstein-Friesian Mexican cattle. These proteins are involved in inflammatory/immunomodulatory responses to infections and may provide a novel avenue of research to determine the mechanisms of protection against bTB. Using validated serum biomarkers to identify and further enhance the resistance of cattle to bTB implemented as part of bTB control strategies could help eventually eradicate bTB from herds by reducing the susceptibility to infection. However, due to the low number of individuals gathered for validation, our results require further investigation to determine the extent to which these results could apply to cattle of different breeds and in different circumstances. In addition, it is clear that the ELISA requires further development and that, ideally, these markers could be evaluated in a multiplex system using perhaps monoclonal antibodies.

## Data Availability Statement

The datasets presented in this study can be found in online repositories. The names of the repository/repositories and accession number(s) can be found below: ProteomeXChange MassIVE MSV000087778.

## Ethics Statement

The animal study was reviewed and approved by Institutional Ethics and Biosafety Committee. Written informed consent for participation was not obtained from the owners because owners gave their verbal consent for sampling of animals previous to slaughter at the abattoir facilities.

## Author Contributions

JM-C, OA-L, BV-R, JG-P, and HE-S contributed to the conception and design of the study. HE-S acquired funding, coordinated the work, performed the statistical analysis, and prepared the final draft of the manuscript. OA-L and HE-S organized the database and conducted laboratory examinations. GL-R and HE-S collected clinical samples and conducted gross examinations. JM-C, OA-L, and HE-S performed the experiments. BV-R, JG-P, GL-R, and HE-S wrote sections of the manuscript. All authors contributed to manuscript revision, read, and approved the submitted version, except OA-L and JM-C who lamentably departed before the final draft was achieved.

## Funding

This research was supported by the projects FOMIXJAL 2009-05-124324 and SEP-CONACYT CB-2009-132068.

## Conflict of Interest

GL-R was employed by the Laboratorios Virbac México SA de CV (a private company of animal health products). The remaining authors declare that the research was conducted in the absence of any commercial or financial relationships that could be construed as a potential conflict of interest.

## Publisher's Note

All claims expressed in this article are solely those of the authors and do not necessarily represent those of their affiliated organizations, or those of the publisher, the editors and the reviewers. Any product that may be evaluated in this article, or claim that may be made by its manufacturer, is not guaranteed or endorsed by the publisher.
