# Supplementary material for: Host Serum Proteins as Potential Biomarkers of Bovine Tuberculosis Resistance Phenotype
Source: Front Vet Sci. 2021 Nov 18;8:734087. doi: 10.3389/fvets.2021.734087 (PMC8637331; doi:10.3389/fvets.2021.734087)
Supplement: Supplementary file 1 [file Data_Sheet_1.pdf]

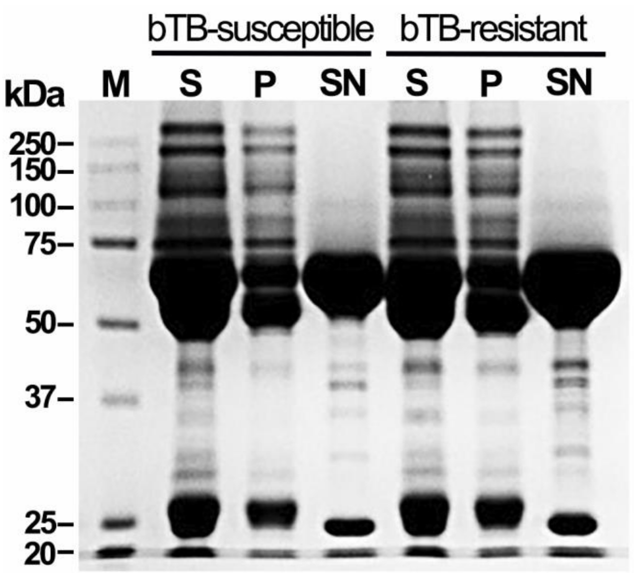

Supplementary Figure 1. Treatment of pooled sera from susceptible or resistant cattle with TCA significantly reduces the presence of high-abundance proteins without dissimilarities between groups. Samples of pooled sera from cattle naturally exposed to *M. bovis* with bTB (susceptible) or without bTB (resistant) were separated on a 10% SDS-PAGE, loaded with 10 µg of protein per well. Samples were untreated sera (S) or the pellet (P) or supernatant (SN) of sera treated with 10% TCA in cold acetone; TCA treatment was carried out as described in materials and methods. At the end of the run, the gel was stained with Coomassie blue to visualize proteins. M, protein molecular weight marker.

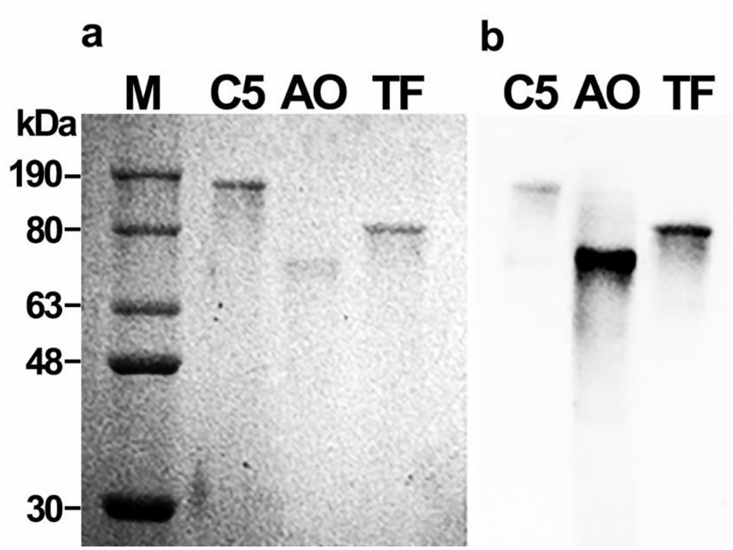

Supplementary Figure 2. SDS-PAGE (12.5%) under reducing conditions of complement component 5 (C5), plasma amine oxidase (AO) and bovine serum transferrin (TF), obtained to produce rabbit polyclonal antibodies for in-house ELISAS, as described in materials and methods. After staining with Coomassie brilliant blue R-250 (**a**), purified material showed unique bands corresponding to molecular weight of proteins. Immunoblotting of transferred proteins to a PVDF membrane (**b**) revealed by using each rabbit polyclonal antibody (diluted 1:200) against its corresponding protein. M, protein molecular weight marker.

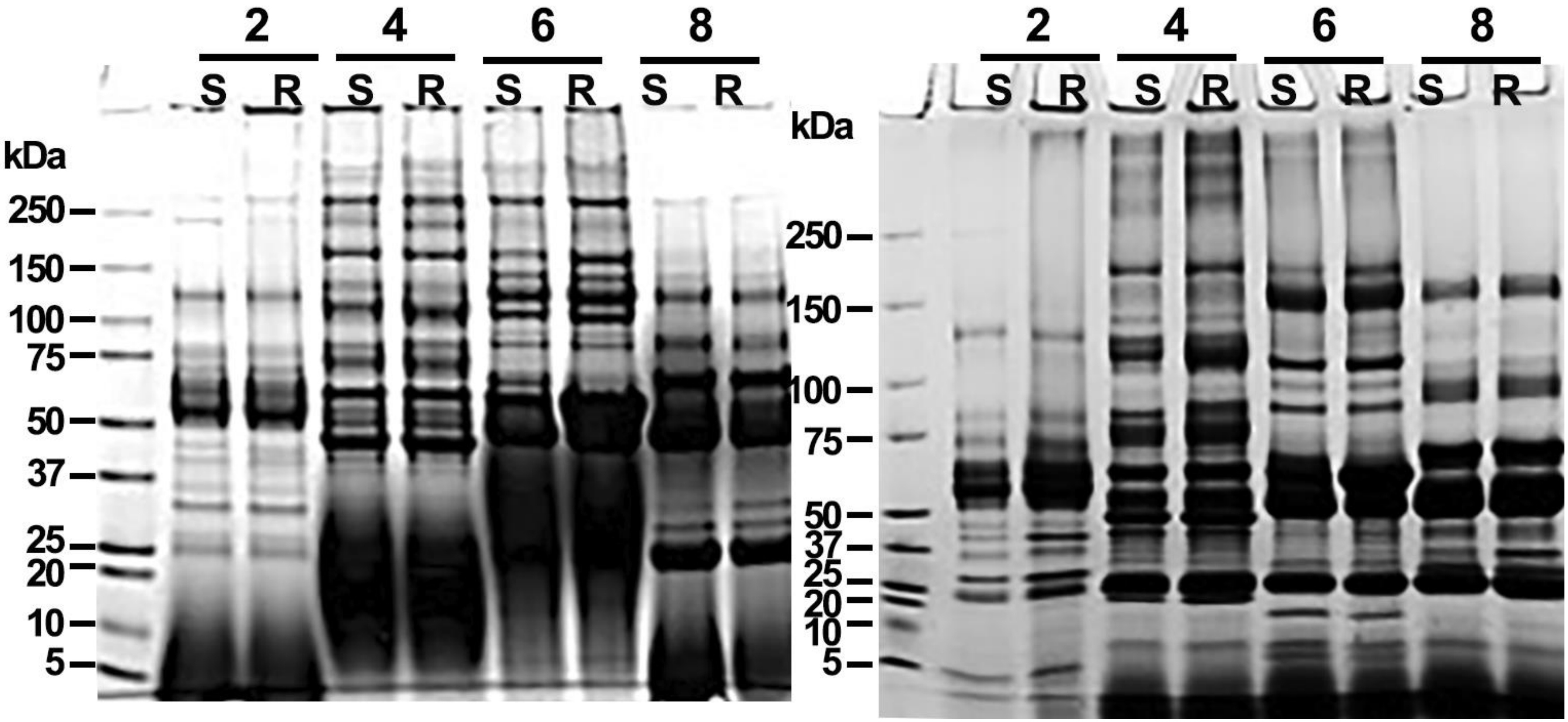

Supplementary Figure 3. Replicas of Coomassie blue stained SDS-PAGEs (4-20% gradient) of single offgel serum fractions (2, 4 6, and 8), paired by susceptible (S) and resistant (R) cattle samples.

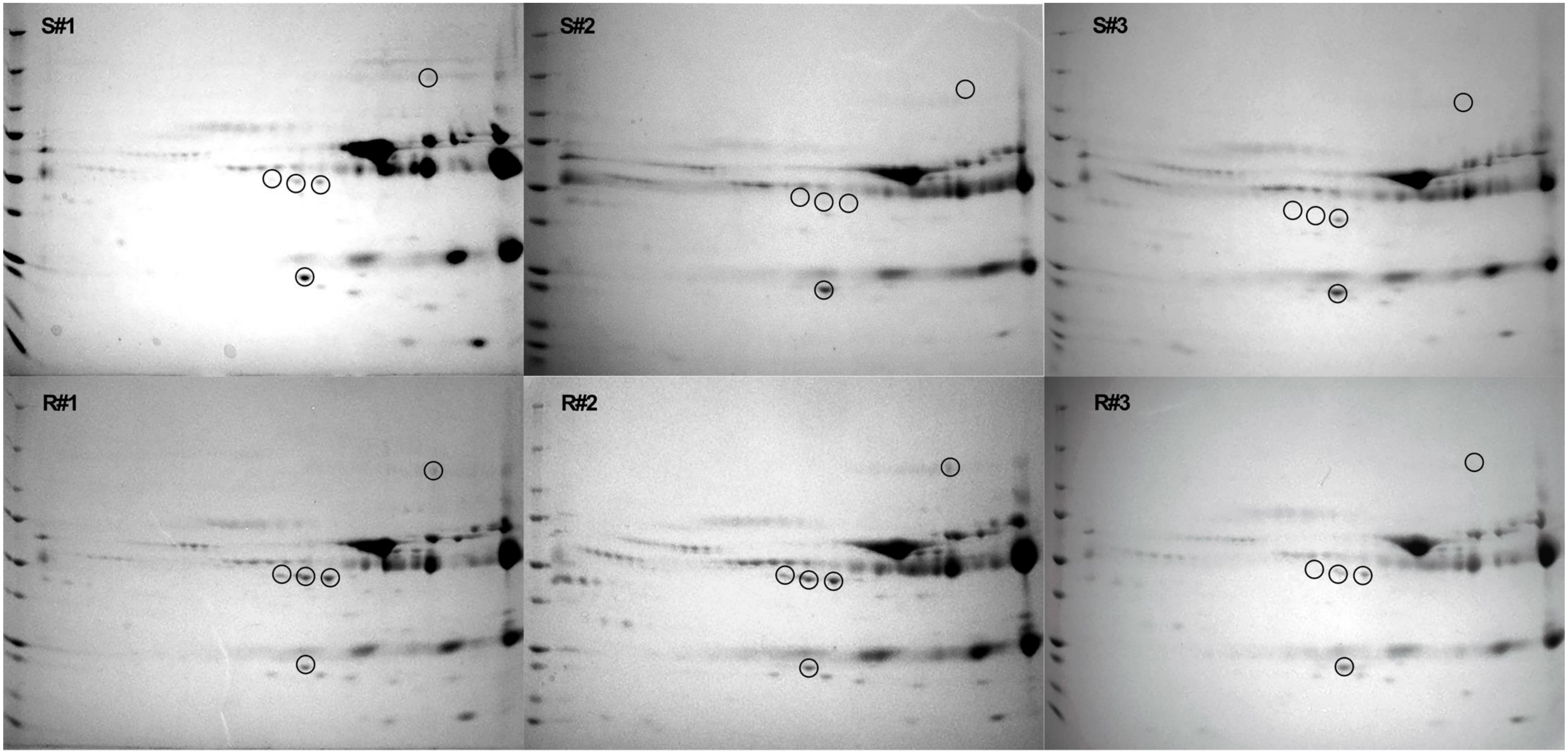

Supplementary Figure 4. Replicas of 2D-GE of proteins from selected offgel fractions of pooled sera from cattle with bTB (S#1, S#2, and S#3) and cattle resistant to bTB (R#1, R#2, and R#3).

Supplementary table 1. Information generated to identify proteins and/or peptides

Spots no.	Peptides matched/ sequence coverage	Mascot score[c]	Protein name Accession no. Theoretical mass (kDa)/pI	Observed mass	Mr(expt)	Mr(calc)	Delta mass	Miscleavages	Peptide/protein	Expect	Match Rank	Peptide sequence (peptide modification)
1	5/4%	87	Complement component 5 A0A0F6QMJ3/ F1MY85 188.677/6.20	447.7401	893.4656	893.4759	-0.0103	0	39	0.036	1	K.YGVWTIR.A
				674.3640	1346.7134	1346.7194	-0.0060	0	65	7.6e-05	1	K.FQNSATLTIQPK.Q
				585.9764	1754.9074	1754.9203	-0.0129	0	39	0.024	1	K.DSFDHLVGGIPVTLSAK.T
				791.3636	2371.0690	2371.0863	-0.0173	0	49		1	K.CGNQLEVHLSPETDAYSPGQR.V + [+71.0371 at N-term C]
				879.0996	2634.2770	2635.2930	-1.0160	1	39		1	R.DKLSDSAYQSINIPVTQDMVPSAR.L + [+0.9970 at S6]
2	8/12%	209	Amine oxidase E1BJN3 76896/5.69	469.2631	936.5116	936.5141	-0.0024	0	54	0.00096	1	K.AAALAHLDR.G
				496.7719	991.5292	991.5451	-0.0158	0	47	0.0058	1	R.YQLAITQR.K
				501.7703	1001.5260	1001.5328	-0.0067	0	65	0.00012	1	K.LLTMNSAPR.G
				622.3128	1242.6110	1242.6245	-0.0134	0	46	0.0062	1	K.ALDPADWTVQK.V
				778.8709	1555.7272	1555.7341	-0.0068	0	39	0.025	1	R.EYLDIDQMIFNR.E
				677.0103	2028.0091	2028.0276	-0.0185	1	80	1.6e-06	1	R.KQLETEEQAAFPLGGASPR.Y
				679.6876	2036.0410	2036.0592	-0.0182	0	35	0.046	1	K.SQVPPGPTPPLQFHPQGPR.F
				679.6877	2036.0413	2036.0592	-0.0179	0	51	0.0011	1	K.SQVPPGPTPPLQFHPQGPR.F
3	17/24%	250	Serotransferrin, plasma transferrin Q29443 77.689/6.75	530.7604	1059.5062	1059.5171	-0.0109	0	48	0.0047	1	R.SAGWNIPMGK.L
				538.7609	1075.5072	1075.5121	-0.0048	0	(41)		1	R.SAGWNIPMGK.L + [+15.9949 at M8]
				583.7941	1165.5736	1165.5801	-0.0065	1	48		1	K.KENFEVLCK.D + [+57.0215 at C8]
				656.3274	1310.6402	1310.6466	-0.0064	0	62	0.00015	1	K.ELPDPQESIQR.A
				668.3506	1334.6866	1334.6904	-0.0038	0	68		1	R.ILESGPFVSCVK.K + [+57.0215 at C-term K]
				675.3597	1348.7048	1348.7061	-0.0012	0	(57)		1	R.ILESGPFVSCVK.K + [+71.0371 at C10]
				695.3358	1388.6570	1388.6684	-0.0114	0	68	4e-05	1	K.TSDANINWNNLK.D

				698.8293	1395.6440	1395.6526	-0.0086	0	76		1	K.CLMEGAGDVAFVK.H + [+57.0215 at N-term C]
				535.6019	1603.7839	1603.7995	-0.0156	0	37	0.035	1	K.DNPQTHYYAVAVVK.K
				547.3096	1638.9070	1639.7590	-0.8521	0	54	0.00065	1	K.HSTVFDNLPNPEDR.K
				572.6282	1714.8628	1714.8890	-0.0262	1	76	4.7e-06	1	K.LYKELPDPQESIQR.A
				586.6191	1756.8355	1756.8533	-0.0178	0	40	0.019	1	K.DKPDNFQLFQSPHGK.D
				590.2830	1767.8272	1767.8540	-0.0268	1	56	0.00041	1	K.HSTVFDNLPNPEDRK.N
				922.9387	1843.8628	1842.8822	0.9807	0	72		1	K.GEADAMSLDGGYLYIAGK.C + [+13.0316 at S7]
				632.0094	1893.0064	1892.0408	0.9656	2	42		1	K.DLLFKDSADGFLKIPSK.M + [-0.9840 at N-term D]
				808.7114	2423.1124	2423.1250	-0.0126	0	44		1	K.SVDDYQECYLAMVPSHAVVAR.T + [+71.0371 at C8]
				638.8050	2551.1909	2551.2199	-0.0290	1	50		1	R.KSVDDYQECYLAMVPSHAVVAR.T + [+71.0371 at C9]
4	4/12%	124	Haptoglobin G3X6K8 41.954/7.10	490.7423	979.4700	979.4876	-0.0175	0	53	0.0013	1	R.VGYVSGWGR.N
				544.8051	1087.5956	1087.6026	-0.0070	0	68	4.6e-05	1	K.VTSILDWVR.K
				673.3244	1344.6342	1344.6384	-0.0041	0	41		1	K.SCAVAEYGVYVK.V + [+57.0215 at N-term S]
				657.6865	1970.0377	1970.0585	-0.0208	1	67	2.7e-05	1	K.NQLVEVEKVVLHPDHSK.V
